# PAMAM-Lys, a Novel Vaccine Delivery Vector, Enhances the Protective Effects of the SjC23 DNA Vaccine against *Schistosoma japonicum* Infection

**DOI:** 10.1371/journal.pone.0086578

**Published:** 2014-01-30

**Authors:** Xiaoting Wang, Yang Dai, Song Zhao, Jianxia Tang, Hongjun Li, Yuntian Xing, Guoli Qu, Xinsong Li, Jianrong Dai, Yinchang Zhu, Xueguang Zhang

**Affiliations:** 1 Jiangsu Stem Cell Key Laboratory, Institute of Medical Biotechnology, Medical College of Soochow University; Jiangsu Institute of Clinical Immunology, The First Affiliated Hospital of Soochow University, Suzhou, China; 2 Jiangsu Institute of Parasitic Diseases, Key Laboratory on Technology for Parasitic Disease Prevention and Control, Ministry of Health, Jiangsu Provincial Key Laboratory on Molecular Biology of Parasites, Wuxi, Jiangsu, China; 3 Biomaterials and Drug Delivery Laboratories, School of Chemistry and Chemical Engineering, Southeast University, Nanjing, China; University of Melbourne, Australia

## Abstract

**Background:**

Schistosomiasis japonica remains a major public-health concern in China. Praziquantel-based chemotherapy effectively reduces both infections and intensity; however, it can not prevent re-infection. Furthermore, there is an increasing concern about praziquantel resistance following long-term repeated use of the drug in endemic areas. Therefore, development of a schistosomiasis vaccine, as a strategy to prevent and control schistosomiasis japonica, has been given high priority. The present study was conducted to develop PAMAM dendrimers as a novel vaccine delivery vector for a schistosomiasis japonica DNA vaccine and evaluate its ability to enhance protective effects against *Schistosoma japonicum* infection.

**Methodology/Principal Findings:**

Lysine was used to modify 4.0G PAMAM, and the modified product PAMAM-Lys was synthesized. PAMAM-Lys showed both high transfection and low cytotocity for gene delivery in vitro. DNA vaccines combined with PAMAM-Lys produced higher level of protection compare with naked DNA vaccines against *S. japonicum* infection in a mouse model. Futhermore,antibodies from mice immunized with PAMAM-Lys combined DNA vaccines were significantly higher than those of mice immunized with the naked DNA vaccines. The PAMAM-Lys vector elicited a predominantly IgG2a antibody response and a tremendously increase in the production of IL-2 and IFN-γ.

**Conclusion/Significance:**

Lysine-modified PAMAM-Lys is an excellent vector. PAMAM-Lys may enhance the immunoreactivity of DNA vaccine and increase the protective effect of the SjC23 DNA vaccine against *S. japonicum* infection.

## Introduction

Schistosomiasis remains a major public health problem throughout the world, with more than 200 million people in 76 countries or regions from Africa, Asia and South America being affected, with an additional 779 million individuals at risk of infection [1]–[3]. In China, schistosomiasis japonica is one of the four key infectious diseases [4] that have been given priority control by the central government. Currently, there are more than 286,000 people infected with the parasite, with 238 million at risk of infection [5]. Despite numerous strategies that have been devised to combat this infectious disease, including the use of chemotherapeutic drugs, such as praziquantel, schistosomiasis still cannot be effectively controlled [6]. It is generally agreed that chemotherapy has certain limitations and drug resistance hampers its effectiveness [7]–[8]. Furthermore, re-infection occurs frequently in endemic areas. Therefore, development of effective vaccine is urgently needed to control and prevent the disease.

With the discovery of potential protective antigens and improved understanding of immune mechanisms for the control of schistosomiasis infection, the development of subunit-based vaccines may be possible. Several potential protective antigens from *S. japonicum* have been reported and used for vaccine development. Some of them have been recommended by WHO/TDR as vaccine candidates, including glutathione S-transferase (Sj26GST) [9], [10], triose-phosphate isomerase (SjTPI) [11]–[13], paramyosin (Sj97) [14], [15], fatty acid binding protein (FABP, Sj14) [16], and 23 kDa membrane protein (Sj23) [10], [12], [16], [17]. Such antigens have been shown to produce partial protection in the mouse model when used as subunit-based vaccines, such as peptide vaccines, recombinant protein vaccines, and DNA vaccines [18], [19]. However, most of these antigens only produce worm reductions of less than 40% in mouse models [14], [15], [17], [20]. Although partial protection may reduce the pathogenesis, morbidity, transmission rates [21], and improve the control of schistosomiasis when combined with praziquantel treatment in humans and livestock [22], [23], it is also important to improve the protective efficacy for an independent prophylactic vaccine.

DNA vaccination was introduced in 1990 when it was demonstrated that protein expression could be induced upon direct intramuscular injection of plasmid DNA into myocytes [24]. The advantages of DNA vaccines over traditional, attenuated or subunit vaccines are the low cost of production, thermal stability, and their ability to induce a wide variety of long-lived cellular and humoral immune responses [25]. In our laboratory, the coding region for *S. japonicum* (Chinese mainland strain) 23-kDa membrane protein (SjC23) was cloned into the eukaryotic expression plasmid, pcDNA3.1, as a DNA vaccine vector. Several different research groups have shown that each of these DNA vaccines induces partial protection in animals, with worm reductions ranging from 20% to 50%, depending on the animal species challenged and the group performing the study [26]–[28].

It has been reported that DNA vaccination, using unformulated plasmid DNA (pDNA), shows low gene transfer efficiency in the host cell and hence, low antigen expression [29]. Recently, cationic polymers carriers, such as polyamidoamine (PAMAM) dendrimers, have been used to deliver pDNA. PAMAM carriers bind the pDNA electrostatically and condense it into positively charged nanoparticles that are more easily taken up by host cells. Furthermore, they protect pDNA against extracellular nucleases [30]. Several studies have already shown that PAMAM dendrimers can enhance the transfection efficiency leading to improved gene expression *in vitro* and *in vivo* [31]. Furthermore, PAMAM conjugated with lysine residues was previously reported to show enhanced transfection efficiency. Therefore, we examined if we could further improve the current DNA vaccine platform to enhance pDNA delivery into the host cells. In this study, we developed lysine-modified PAMAM dendrimers as a novel vaccine delivery vector for a schistosomiasis japonica SjC23 DNA vaccine and evaluated its ability to enhance protective effects against *S. japonicum* infection.

## Materials and Methods

### *Schistosoma Japonicum* Cercaria

*Oncomelania hupensis* snails, infected with *S. japonicum* Chinese strain were obtained from Jiangsu Institute of Parasitic Diseases, China. Cercariae of *S. japonicum* were collected from infected snails for subsequent experiments.

### Construction of PJW4303/SjC23 Vaccine

A pair of primers (P1: 5′-GC *GCTAGC* **ATG** GCG ACT TTG GGTACT-3′; P2: 5′-GC *GGATCC* AAC ATT CTG ATA ATCG-3′) were designed and synthesized based on the gene sequence of the 23 kDa membrane protein from *S. japonicum* Chinese strain (SjC23) containing *Nhe* I and *BamH* I restriction sites. The SjC23 gene was amplified with the pcDNA3.1-SjC23 as the template, and the amplified product was cloned into the polyclonal region of PJW4303 (kindly presented by Dr. Shan Lu). Then the recombinant plasmids were identified using double-digestion and DNA sequencing.

### Construction of PJW4303/SjC23-mHSP70 Vaccine

Two primers (P3: 5′-GC *CTAGC* ATG GCG ACT TTG GGT-3′; P4: 5′-GC *GGATCC* ATT CTG ATA ATCG-3′) were designed and synthesized based on the gene sequence of SjC23 containing *Nhe* I and *BamH* I restriction sites at the 5′ terminal and removal of the termination codon. The fragment was amplified from pcDNA3.1-SjC23 using a PCR technique and then sub-cloned into PJW4303 to construct PJW4303-SjC23. Another two primers (P5: 5′-GC *GGATCC* ATG GCC AAG AAC ACG-3′; P6: 5′-GC *CTCGAG* CTA ATC CAC CTC CTC-3′)were designed and synthesized based on the gene sequence of mHSP70 containing *BamH* I and *Xho* I restriction sites. The fragment was amplified from PVAX1-mHSP70 (kindly gifted by Dr. Shan Lu) and then sub-cloned into PJW4303-SjC23 to construct the PJW4303-SjC23-mHSP70 vaccine.

### Mass Production of the DNA Vaccine

Mass production of the plasmid PJW4303, PJW4303-SjC23 and PJW4303-SjC23-mHSP70 DNA vaccines was conducted according to manufacturer’s instructions for QIAGEN-2500. The concentration and purity was detected by the BECKMAN DU-600 Spectrophotometer. The *A*_260_/*A*_280_ value of the prepared DNA ranged between 1.85 and 1.95. DNA was resuspended in sterile physiological saline (0.9% NaCl, PH 7.4). All DNAs were diluted to 1 mg/ml.

### Synthesis and Characterization of PAMAM-Lys

Condensation was carried out on 4.0G PAMAM (which was stored in our lab) [32]. Fmoc-Lys(Boc)-OH (GL Biochem (Shanghai) Ltd.), Fmoc-protective groups, and Boc-protective groups were removed via chemical reaction. The product was dissolved in water and dialyzed for 12 h at 4°C using a dialysis membrane (Green Bird Technology) with molecular weight cut-off (MWCO) of 14,000. For analysis, the polymer samples were dissolved in ddH_2_O containing 3-(trimethylsilyl) propionic-2,2,3,3-d4 acid sodium salt as an internal reference. The product was collected under a freeze-dry conditions, and white powder PAMAM-Lys was obtained. ^1^H NMR spectra of the 4.0G PAMAM and PAMAM-Lys were obtained using a Bruker Advance −300 NMR spectrometer (399 Hz).

### Gel Retardation Assay

To determine the most appropriate charge ratio(*R*_+/−_) of plasmid DNA and PAMAM-Lys, PAMAM-Lys/plasmid complexes were prepared at various *R*_+/−_ ratios ranging from 0.5 to 10. After 30 min of incubation at room temperature for complex formation, the samples were electrophoresed on a 1% (w/v) agarose gel, stained in an ethidium bromide solution (0.5 mg/mL), and analyzed on a UV illuminator to show the location of the DNA.

### Preparation of DNA/PAMAM or PAMAM-Lys Complex Formation

Since PAMAM has a positive charge and DNA has a negative charge in aqueous solution, PAMAM and DNA can form a complex with electrostatic effects in physiological conditions. PAMAM-Lys/plasmid and PAMAM/plasmid complexes at a most appropriate charge ratio were incubated for 30 min at room temperature for *in vivo* transfection and DNA vaccine development.

### Transmission Electron Microscopic Observation of the PAMAM-Lys/plasmid Complexes

Morphology of PAMAM-Lys/plasmid complex was observed under a transmission electron microscope (TEM). Approximately 10 µl of sample was dropped onto the Cu grid; after absorption for 2 min, blotting with filter paper, and open-air drying, it was observed under a TEM. The voltage was 100 kV and the amplifications of TEM were 40,000, 60,000 and 80,000.

### Cytotoxicity Assay of PAMAM

The cytotoxicity of the polymers was measured by MTT (3-(4,5-Dimethylthiazol-2-yl)-2,5-diphenyltetrazolium bromide) assay. Human kidney transformed cells 293T (China Center for Type Culture Collection, CCTCC) were maintained in DMEM medium with 10% FBS at 37°C in 5% CO_2_. 293T cells were placed in a 96-well tissue culture plate at 10^4^ cells/well in DMEM medium containing 10% FBS. Cells achieving 70–80% confluence after 24 h were exposed to polymer solutions with various concentrations for 48 h. Then, 50 µl stock solution of MTT (2 mg/ml in PBS) was added to each well. After 4 h of incubation at 37°C, each medium was removed and 150 µl of DMSO was added to each well to dissolve the formazan crystals formed by proliferating cells. Absorbance was measured at 570 nm using a microplate reader (Anthos Zenyth340). All the procedure repeat 5 times and results expressed as mean ± standard deviation. Cell viability is recorded as a percentage relative to untreated control cells.

### Expression of PAMAM-Lys/PJW4303-SjC23 in vitro

To determine expression and transfection efficiency of the PAMAM-Lys/SjC23, the SjC23 gene was inserted upstream of the report gene, GFP, in plasmid pEGFP-N3. The constructed plasmid pEGFP-N3-SjC23 was identified using double-digestion and DNA sequencing. 293T cells were seeded at a density of 5×10^4^ cells/well in a 6-well plate in a DMEM medium containing 10% FBS and grown to reach 70%–80% confluence prior to transfection. Before transfection, the medium was exchanged with fresh serum-free medium. The cells were treated with polyplex solution containing 2 µg of plasmid DNA at different charge ratios for 4 h at 37°C. After incubation for 4 h at 37°C and 5% CO_2_, complexes were removed, cells were rinsed again with PBS and complete culture medium was added. Naked pDNA and complexes with lipofectamin2000 transfection reagent (Invitrogen) were used as negative and positive controls, respectively. Measurement of gene transfer efficiency was performed in triplicate. After exchange with fresh serum containing medium, cells were further incubated for 2 days after transfection. At 48 h following transfection, cells were trypsinized and resuspended in PBS. To quantify SjC23 expression, the percentage of transfected cells was determined by measuring EGFP fluorescence (488 nm) using a FACSCalibor flow cytometer (BD Biosciences, Erembodegem, Belgium). All the procedure repeat 3 times, and results are expressed as mean ± standard deviation.

### DNA Vaccine Immunization in Mice

5∼6 weeks old female BALB/c mice were purchased from Shanghai Slac Laboratory Animal Co, Ltd (Shanghai, China) and maintained in specific pathogen-free, environmentally controlled conditions according to standard laboratory chow. A total of 100 mice were divided randomly into 7 groups (N = 14–15 per group); groups were referred to as the control group (physiological saline group), PJW4303 group (Vector group), PAMAM-Lys group, PJW4303-SjC23 group, PAMAM-Lys/PJW4303-SjC23 group, PJW4303-SjC23-mHSP70 group, and PAMAM-Lys/PJW4303-SjC23-mHSP70 group; each mouse in the control group was vaccinated intramuscularly with physiological saline while the others were vaccinated with 100 µg PJW4303, PAMAM-Lys, PJW4303-SjC23, PAMAM-Lys/PJW4303-SjC23, PJW4303-SjC23-mHSP70, or PAMAM-Lys/PJW4303-SjC23-mHSP70. Mice were boosted two times with aforementioned DNA vaccine plasmids using the same dosage and method, every 2 weeks. Serum samples were collected prior to the initial DNA vaccination and at 4 weeks after the final immunization for detection of antibody responses using indirect enzyme-linked immunosorbent assay (ELISA).

### Cytokine Measurement

Two mice were randomly selected from each group and sacrificed three weeks after the 3rd immunization. Spleens were removed, and single-cell suspensions were prepared by producing splenocytes from the two mice from each group for measurement of T cell immune responses. The suspensions were then cultured in triplicate in 96-well plates at 6×10^6^ cells/well in RPMI 1640 medium supplemented with 10% fetal calf serum for 72 h at 37°C with 5% CO_2_ in the presence of rSjC23-LHD protein (large hydrophilic domain) at 5 µg/ml. Cytokine levels, including interleukin-2 (IL-2), IL-4, IL-5, IFN-γ, and tumor necrosis factor (TNF) in supernatant were measured by flow cytometry analysis on the BD FACSCalibur Flow Cytometer using Cytometric Bead Array Mouse Th1/Th2 Cytokine Kit (BD), per manufacturer’s instructions.

### Determination of Antigen-specific Antibody

ELISA was performed to detect SjC23LHD-specific antibody. SjC23LHD, the long hydrophilic domain of SjC23, contains main T and B cell epitopes of SjC23 antigen (the full length of SjC23 cannot be expressed in *E.coli*. Briefly, rSjC23LHD (which was stored in our lab) [33] protein was diluted in 50 mM carbonate buffer (pH 9.6) to 5 µg/ml. 100 µl was then added to each well on 96-well plates and incubated at 4°C overnight for antigen coating. Each plate was washed three times with PBS (pH 7.6) containing 0.05% Tween-20 (PBST), and blocked with 3% (w/v) bovine serum albumin (BSA) in PBS for 3 h at 37°C. The plates were further washed three times with PBST, and then incubated with the mouse immune sera serially diluted in PBS for detection of IgG, IgG1, and IgG2a at 37°C for 1 h. The plates were then washed five times with PBST, followed by incubation with HRP-conjugated goat-anti-mouse IgG, IgG1, and IgG2a (Santa Cruz Biotechnology, Santa Cruz, CA) for 45 min at 37°C. Plates were then washed five times with PBST and were developed with tetramethylbenzidine substrate and read at 450 nm after the reaction was terminated using 2 M sulphuric acid.

### Assessment of Protective Efficacy

All mice were challenged with 40±1 cercariae of *S. japonicum* through abdominal skin penetration 4 weeks after the 3rd immunization. Six weeks post-challenge, mice were sacrificed and perfused to determine adult worm burdens. Mice livers were removed and weighed and then digested overnight with 5% potassium hydroxide (5 ml) at 37°C. Worm and egg reduction rates were calculated as follows: worm reduction rate (%) = (average worm burden in control group-average worm burden in test group)/average worm burden in control group×100%, egg reduction rate (%) = (eggs per gram in control group-eggs per gram in test group)/eggs per gram in control group×100%.

### Statistics

One-way analysis of variance (ANOVA) was used to compare the cytotoxicity of PAMAM the protection efficacy including the percent reduction in worm and egg burdens and the antibody responses. Student’s t test was used to analyze transfection efficiency of the PAMAM and the cytokine measurement. A *p* value of less than 0.05 was considered statistically significant.

### Ethics Approval

This study was approved by the Institutional Review Board (IRB00004221) of Jiangsu Institute of Parasitic Diseases (Wuxi, China). The animal experiment protocol was performed according to administration of lab animals issued by Ministry and Technology (Beijing, China) and approved by Jiangsu Institutional Animal Care and Use Committee (IACUC).

## Results

### Synthesis and Characterization of PAMAM-Lys

**Figure 1:**
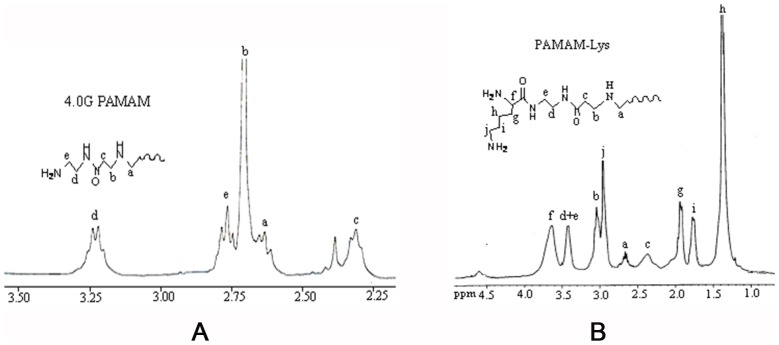
^1^H-NMR data for 4.0G PAMAM and PAMAM-Lys. (A)^1^H-NMR data of 4.0G PAMAM; (B)^1^ H-NMR data of PAMAM-Lys Data show chemical shifts (a,b,c,d,e,f,g,h,I,j) of the terminal unit for 4.0G PAMAM and PAMAM-Lys.

l-lysine-grafted-PAMAM dendrimer (PAMAM-Lys), synthesized via conjugation of l-lysine, was analyzed by ^1^H-NMR spectroscopy. As shown in Fig. 1, chemical shifts of the terminal unit for 4.0G PAMAM were located in 2.64(a), 2.71(b), 2.32(c), 3.23(d), and the Amide N-H near 2.77(e) ppm (Fig. 1A) while chemical shifts of PAMAM-Lys were located in 2.65(a), 3.05(b), 2.38(c), 3.41(d), 3.42(e), 3.62(f), 1.91(g), 1.40(h), 1.78(i), and 2.96(j) ppm (Fig. 1B), which is consistent with previous study [34]. In comparison, the H atom chemical shifts of PAMAM-Lys were similar under the same environment as revealed by ^1^H-NMR. There was no difference of the basic structure however, PAMAM-Lys had one more reaction group layer compared with 4.0G PAMAM, which was considered a more complicated structure than 4.0G PAMAM. The amine number in the outermost layer of PAMAM-Lys was equivalent to that of 5.0G PAMAM.

### Complex Formation Detected by Agarose Gel Electrophoresis Reagent Assay

**Figure 2:**
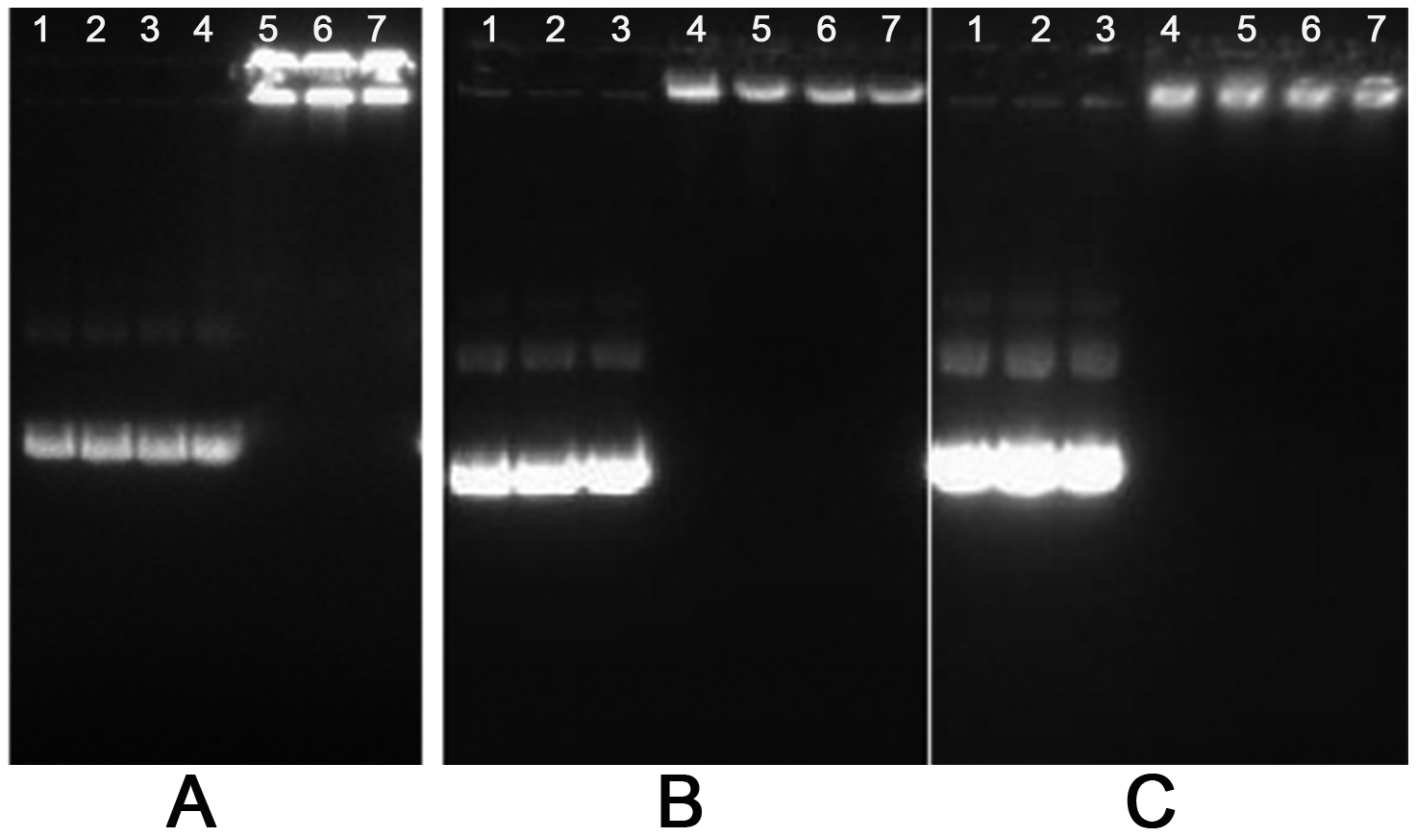
Agarose gel electrophoresis retardation assay of plasmid DNA byPAMAM4.0G (A), PAMAM 5.0G(B), and PAMAM-Lys(C). Plasmid DNA (0.5 µg) only (lane 1); charge ratio of polymer/DNA = 0.5, 1, 2, 4, 8 and 10 (lanes 2, 3, 4, 5, 6, and 7, respectively).

To assess the formation of dendrimer/DNA complexes, agarose gel electrophoresis of the complex was performed at different charge ratios (Fig. 2). The results showed that the plasmid DNA showed complete retardation at a charge ratio of two 4.0G PAMAM or four with 5.0G PAMAM and PAMAM-Lys, which may be explained by the fact that the layer number in all and amine number in outermost of PAMAM-Lys were equivalent to that of 5.0G PAMAM.

### Size of Plasmid DNA and PAMAM-Lys Complexes

**Figure 3:**
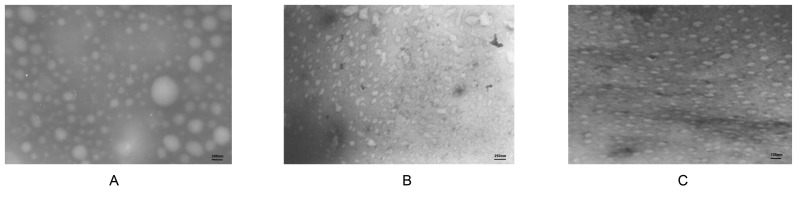
Size of plasmid DNA and PAMAM-Lys complexes. SEM photograph of plasmid DNA (x50,000) (A) compound PAMAM G4.0/DNA (R_+/−_ = 2; ×40,000) (B) and compound PAMAM-Lys/DNA (R_+/−_ = 4; ×80,000) (C).

Through microscopic examination, PJW4303 showed some free plasmids with irregular sizes at a magnification of ×50,000, with diameters between 100 and 300 nm (Fig. 3A). When 4.0G PAMAM bound with DNA, at a charge ratio of 2, the diameter of the plasmid decreased, and the particle size of the complex tended to be even smaller under the microscope at a magnification of ×40,000 (Fig. 3B). The PAMAM-Lys/DNA complex (at a charge ratio of 4) produced particles with a regular arrangement observed under the microscope at a magnification of ×80,000 (Fig. 3C), with particle diameters of between 50 and 100 nm. These results demonstrate that interactions between PAMAM dendrimers and DNA with negative charge neutralized the negative charge carried by the DNA, leading to a decrease size of the DNA structure.

### Cytotoxicity of PAMAM-Lys

**Figure 4:**
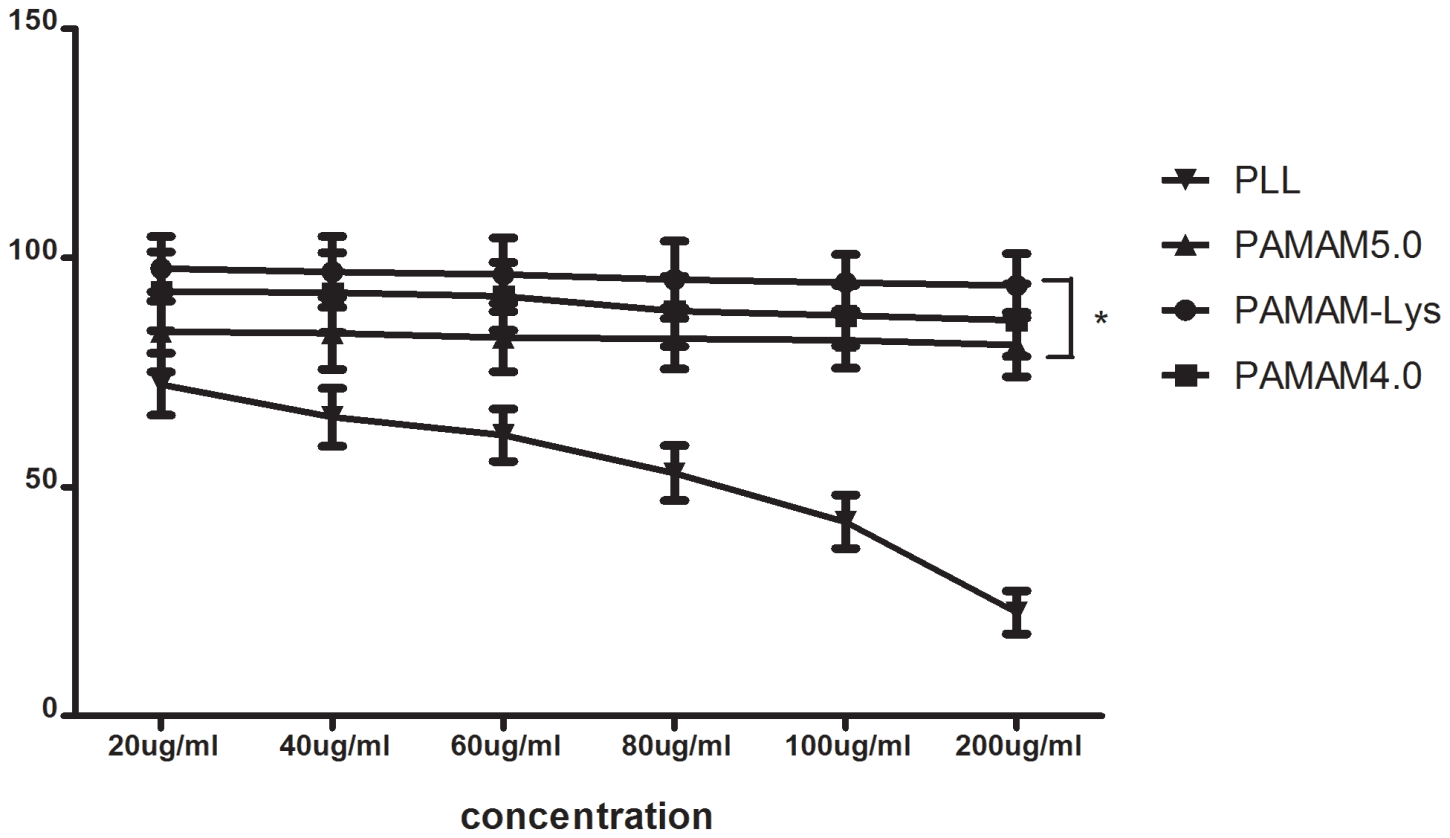
Cytotoxicity assay in 293T cells PLL (▪), PAMAM5.0G (•), PAMAM-Lys (▾), PAMAM4.0 (▴). Each DNA complex was incubated with the cells for 24± standard deviation of the number of live cell every 100 cells. *p<0.05.

The cytotoxicity of PAMAM was examined on 293T cell lines by MTT assay, while poly-l-lysine (PLL) served as a control. As shown in Fig. 4, 293T cell viability after treatment with PLL was 72.4% at 20 µg/mL. As PLL concentration increased, cell viability decreased; PLL at a concentration of 200 µg/ml decreased cell viability to 17%. However, the PAMAM dendrimer (including 4.0G PAMAM, 5.0G PAMAM and PAMAM-Lys) exhibited 80%–90% cell viability consistently at the 20 µg/mL to 200 µg/mL concentration range (Fig. 4). In general, cytotoxicity of gene delivery carriers is known to arise from the accumulation of non-degraded and nondischarged polymers with large molecular weight and charges. This result showed that cytotoxicity of the dendrimer was minimal and lysine conjugation to 4.0G PAMAM dendrimer did not pose a negative effect in its cytotoxicity. Cell viability of PAMAM-Lys was also found to be higher than that of 5.0G PAMAM (*p<0.05*).

### Transfection Efficiency on 293T Cell Lines

**Figure 5.Transfection:**
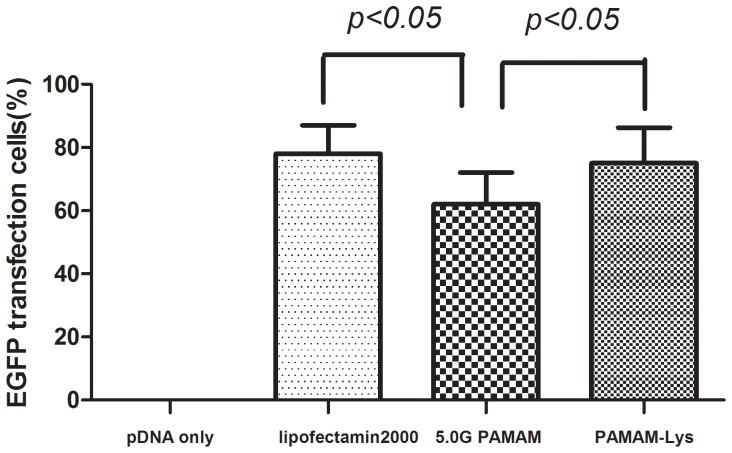
efficiency of PAMAM-Lys - 5.0G PAMAM complex determined by flow cytometry in 293T cells. Each data point represents the mean ± standard deviation (n = 3). At 48 h following transfection, cells were collected and the percentage of transfected cells was determined by measuring EGFP fluorescence (488 nm).

The transfection efficiency of PAMAM-Lys reached 75%, which was higher than that of 5.0G PAMAM (60%)(*p<0.05*) (Fig. 5). Lysine conjugation to PAMAM dendrimer led to highly enhanced transfection efficiency, suggesting that PAMAM-Lys possessed high cell permeability and transfection potency.

### PAMAM-Lys Enhances DNA Vaccine-induced Specific Humoral Responses in Mice

**Figure 6:**
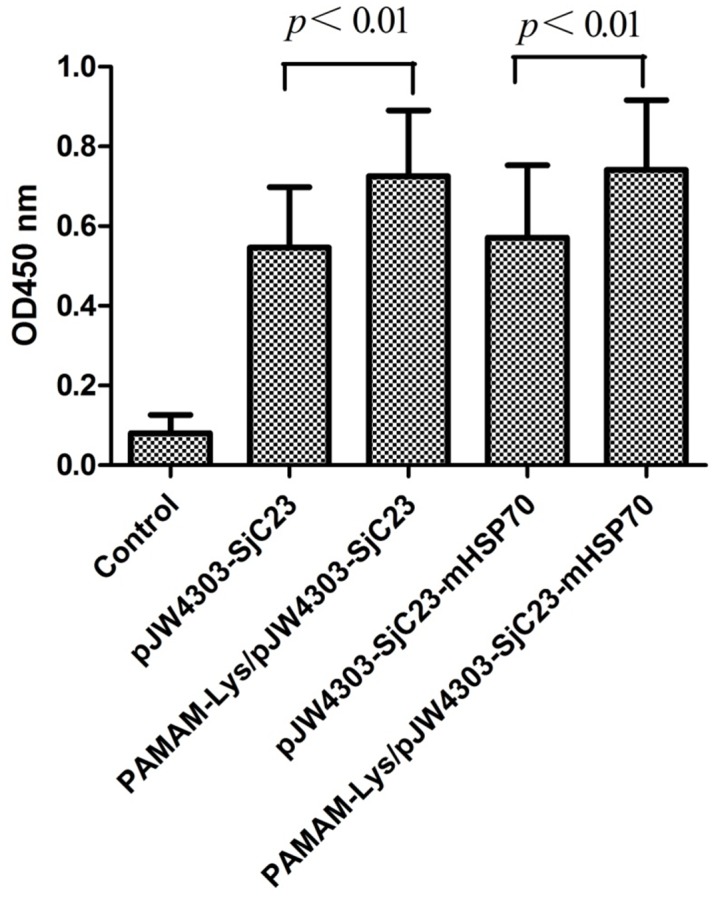
SjC23-specific IgG antibody responses induced by DNA vaccines and empty vector by intramuscular (i.m) immunization. Data show the average OD values of each group with standard deviations at 1∶100 serum dilution. The serum samples were collected at 4 weeks after the 3^rd^ DNA immunization prior to challenge. Statistical differences are indicated by *p* values.

The relative immunogenicities of PAMAM-Lys/DNA vaccines and naked DNA vaccines were evaluated in mice. At 4 weeks after the 3rd DNA immunization by intramuscular injection, SjC23-specific antibody responses in mouse sera were measured against the recombinant SjC23 antigen by ELISA. The PAMAM-Lys/PJW4303-SjC23 DNA vaccine elicited higher levels of anti-SjC23 IgG responses than observed with the naked PJW4303-SjC23 DNA vaccine. In addition, the PAMAM-Lys/PJW4303-SjC23-mHSP70 DNA vaccine produced a higher level of anti-SjC23 IgG responses compared with the naked PJW4303-SjC23-HSP70 DNA vaccine (Fig. 6). The results showed that DNA vaccine/PAMAM-Lys complex induced higher specific humoral responses than observed with the naked DNA vaccine.

### PAMAM-Lys/PJW4303-SjC23 DNA Vaccine Improves Th1-type Antibody and Cytokine Responses

**Figure 7:**
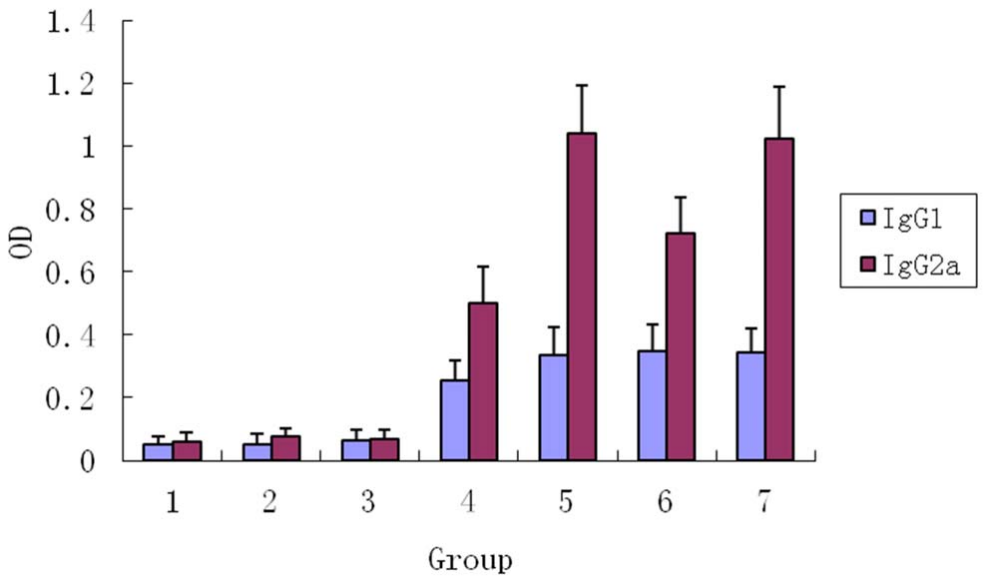
SjC23-specific IgG1 and IgG2a antibody response induced by DNA vaccines and empty vector by intramuscular (i. m) immunization. Data show the average OD values of each group with standard deviations at 1∶100 serum dilution. Statistical differences are indicated by *p* values. 1. Control, 2. pJW4303, 3. PAMAM-Lys, 4. pJW4303-SjC23, 5. PAMAM-Lys/pJW4303-SjC23, 6. pJW4303-SjC23-mHSP70, 7. PAMAM-Lys/pJW4303-SjC23-mHSP70.

**Figure 8:**
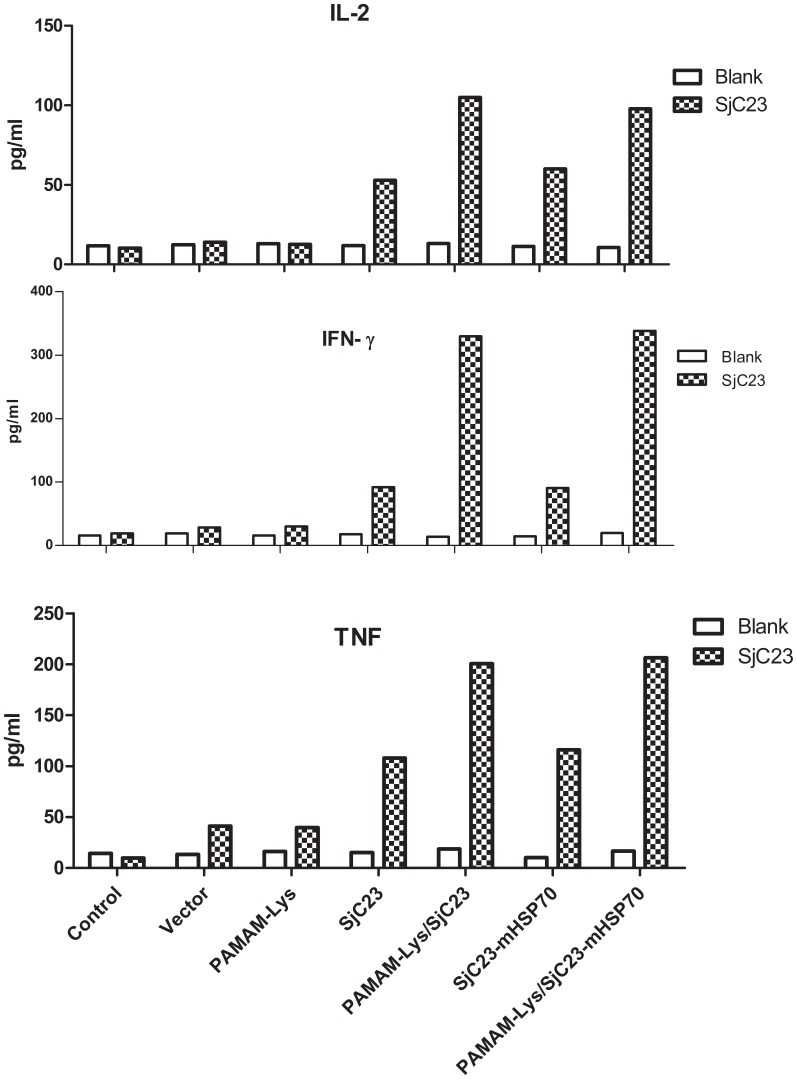
SjC23-specific cytokine responses in splenocytes induced by DNA vaccines,PAMAM-lys, PAMAM-lys/vaccines and empty vector. Data show IL-2, IFN–γ and TNF responses with SjC23 stimulation or blank control. The splenocytes were collected at 3 weeks after the 3rd DNA immunization.

Further analysis demonstrated that increased antibody responses by PAMAM-Lys/DNA vaccine were mainly caused by increased IgG2a (*P*<0.001) and not IgG1 (Fig. 7). Therefore, our data suggest that the PAMAM-Lys/DNA vaccine complex was more effective in eliciting Th1-type antibody responses. Mice that received the empty DNA vaccine vector PJW4303 and PAMAM-Lys immunization maintained low background responses without any significant antibody responses against SjC23. This finding was further confirmed by the measurement of cytokines in immunized animals. At 4 weeks after the 3rd DNA immunization by intramuscular injection, splenocytes from immunized mice in each vaccine group were harvested. The cells were then stimulated with recombinant SjC23-LHD protein (10 µg/ml) or media alone (negative control). Concentrations of Th1 cytokines (IL-2, IFN-γ and TNF) in 72-h culture supernatant were measured. The results indicated that splenocytes from animals that received the PAMAM-Lys/DNA vaccine produced higher levels of Th1 cytokines when compared to cells harvested those who received the naked DNA vaccine (Fig. 8). Cells stimulated with mock medium or those from mice immunized with the empty vector did not show significant levels of cytokine responses. Th2 cytokines, IL-4 and IL-5, were also measured, but these Th2 cytokines were below detectable levels in supernatant fluids collected 72 h after culture.

### PAMAM-Lys SjC23 Enhanced the Protective Immunity against *Schistosoma Japonicum* Challenge

To test the protection efficacy induced by PAMAM-Lys**/**DNA vaccines and naked DNA vaccines, immunized mice were challenged with *S. japonicum* cercariae by abdominal skin penetration, 4 weeks after the 3^rd^ DNA immunization via intramuscular injection. Worm and egg burdens were measured in mouse vena mesenteria and liver tissue at 6 weeks after the infection. Numbers of adult worms and eggs were significantly reduced in the DNA vaccine groups when compared to controls. The reduction rates of adult worms and eggs were 35.33% and 43.61%, respectively (Table 1 and 2). Furthermore, mice receiving PAMAM-Lys/SjC23 DNA vaccine had reduction rates of 46.44% and 61.95% for adult worms and eggs respectively, both significantly higher than rates observed following immunization with the SjC23 DNA vaccine(*P<0.05*). Furthermore, reduction rates of adult worms and eggs in mice vaccinated with PAMAM-Lys/SjC23-mHSP70 DNA vaccine were significantly higher than rates observed in mice vaccinated with pJW4303-SjC23-mHSP70. These results indicate that protection in PAMAM-Lys/DNA vaccine group was significantly improved when compared to mice that received naked DNA vaccine (*P*<0.01). These results showed that the complex PAMAM-Lys/DNA vaccine not only elicited higher antibody and Th1 cytokine responses but also achieved higher efficacy against *S. japonicum* infection.

**Table 1:** Comparison of recovered adult worms in each group.

Group	No. of mice	Average no. of worms ( 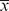 ±s )	worm reduction rate (%)	*P* value
PAMAM-Lys/pJW4303-SjC23-mHSP70	12	15.83±3.15	48.30	<0.01
pJW4303-SjC23-mHSP70	12	20.42±4.79	33.31	<0.01
PAMAM-Lys/pJW4303-SjC23	12	16.40±2.41	46.44	<0.01
pJW4303-SjC23	12	19.80±4.11	35.33	<0.01
PAMAM-Lys	13	29.43±3.89	1.9	>0.05
pJW4303	13	30.00±5.06	0.87	>0.05
Control	13	30.62±4.82	–	–

Data are presented as mean ±SD, n = 12–13;

Worm reduction rate of each group is calculated by comparing with control group.

**Table 2:** Comparison of recovered liver eggs in each group.

Groups	No. of mice	Average no. of eggs ( 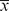 ±s )	Egg reduction rate (%)	*P* value
PAMAM-Lys/pJW4303-SjC23-mHSP70	12	46000±12308	59.30	<0.01
pJW4303-SjC23-mHSP70	12	59167±10456	47.65	<0.01
PAMAM-Lys/pJW4303-SjC23	12	43000±10115	61.95	<0.01
pJW4303-SjC23	12	63727±11387	43.61	<0.01
PAMAM-Lys	13	112715±20148	0.28	>0.05
pJW4303	13	106454±26742	5.81	>0.05
Control	13	113026±24364	–	–

Data are presented as mean ±SD, n = 12–13.

Egg reduction rate of each group is calculated by comparing with control group.

### Immune Responses Induced by DNA Vaccine Expressing SjC23-mHSP70 Fusion Antigen

According to previous reports, mHSP70 may enhance the immunogenicity of DNA vaccination in mice [35]. To test its effects on the SjC23 DNA vaccine, mHSP70 was fused to the C-terminus of SjC23 to express a SjC23-mHSP70 fusion protein, SjC23-mHSP70. Compared to mice that received SjC23 vaccination (PAMAM-Lys/PJW4303-SjC23), mice receiving the SjC23-mHSP70 vaccination (PJW4303-SjC23-mHSP70 or PAMAM-Lys/PJW4303-SjC23-mHSP70) produced similar levels of antibody (Fig. 6), cytokine responses (Fig. 8), and a similar degree of protection (Table 1 and 2) after the 3rd DNA immunization. These data show that the fusion of mHSP70 to SjC23 did not further enhance the immunogenicity of SjC23 DNA vaccines.

## Discussion

Current schistosomiasis japonica control strategies in China are based on chemotherapeutic treatment of infected individuals and eradication of the snail intermediate hosts [36]. In the lake and marshland regions of China, seasonal transmission means that a large percentage of individuals residing in these areas will be re-infected on an annual or frequent basis, thereby requiring constant monitoring of infection and drug treatment with praziquantel. Since praziquantel is the only drug of choice for more than 30 years, annual treatment with the drug provides an opportunity for drug resistance. In other countries, praziquantel resistance has been reported due to the mass chemotherapy programs [37]. Thus, the development of alternative, long-term control measures, such as vaccines, is required. The use of partially protective vaccines administered in conjunction with praziquantel treatment has been considered an ideal control measure for some time. To date, a number of candidate vaccines have been tested in various animal models of schistosomiasis japonica, including several different DNA vaccines (SjFABP, SjGCP, SjGST, SjC23, and SjCTPI). Each of these DNA vaccines provided partial protection against *S. japonicum* infection in mice [38]. The purpose of this study was to determine if administration of partially protective DNA vaccines via PAMAM-Lys dendrimers would significantly elevate vaccine efficacy over that previously reported. Additionally, SjC23, a membrane protein of *schistsoma japonica*, is important for recognition of host, immune system,therefore, we decided to vaccinate mice with target parasite membranes (SjC23) for this study.

Cationic gene carriers have been developed to substitute viral vectors for their avoidance of immunogenecity and oncogenecity. To date, several synthetic and natural cationic polymers have been introduced and tested for their potential applicability to the field of gene delivery [39]. Some cationic polymers showed unexpected adverse effects, such as low transfection efficiencies *in vivo* and inherent cytotoxicity, eventually limiting their use as *in vivo* gene carriers. Polycationic dendrimers have gained increasing attention because of their well-defined structure, protecting DNA from enzymatic degradation as well as easy control of surface functionality for the design of biomedical applications [40]. At present, polyamidoamine (PAMAM) dendrimer, a kind of polycationic dendrimer, has been tested for its potential utility and has exhibited relatively high transfection efficiencies *in vitro*. Furthermore, with the in growth of generation, PAMAM dendrimer showed higher transfection efficiencies, but also higher cytotoxicity [41]. Therefore, one of the major challenges with using PAMAM as a gene delivery system is the need to lower its cytotoxicity. Interestingly, it is known that lysine, essential amino acids for vertebrata, contains positively charged amino acid residues that can combine with negatively charged DNA in aqueous solution. The focus of this paper was to present a new l-lysine-grafted-PAMAM dendrimer (PAMAM-Lys), as a novel non-viral gene delivery vector, which is composed of a backbone of PAMAM dendrimer and the surface of which is covered with basic l-lysine residues. In this study, PAMAM-Lys was prepared and showed significantly better transfection efficiency in 293T cells than observed for native PAMAM. This result showed that, by introducing lysine residues to PAMAM surfaces, gene delivery potency greatly increased in comparison with that of native PAMAM for 293T cells. Furthermore, cytotoxicity of PAMAM-Lys was lower compared to 5.0G PAMAM. These results suggested that PAMAM-Lys meets requirements needed for both high transfection and low cytotocity. The PAMAM-Lys can be used as an effective delivery vector for DNA vaccines and it also can be used as a platform for other gene delivery systems.

Highly effective and safe vaccines are urgently needed for the sustainable control of schistosomiasis. Previous studies have elucidated that high levels of protective immunity can be induced in animals such as mice, rats, cattle, pigs, and rhesus monkeys immunized with radiation-attenuated (RA) schistosoma cercariae [42]. It has been considered the most effective vaccination approach against schistosomiasis in animal models. Further investigations into the mechanisms of protection induced in mice vaccinated with a single dose of RA cercariae indicated that this protective immunity may be mediated by Th1-type immune responses [43].

In this study, predominantly Th1-type antibody and cytokine responses were observed with DNA vaccines. Furthermore, the combination of PAMAM-Lys vector delivery approaches did not change this Th1 dominance, which may contribute to the improvement in protection efficacy following immunization with PAMAM-Lys SjC23DNA vaccines. Thus, PAMAM-Lys as a DNA vaccine vector can facilitate Th1-Type immune responses. In future research, more detailed analysis on the polyfunctionality of the T cell immune responses that induced these Th1-type responses should be included to potentially benefit prospective vaccine design strategies.

In this study, DNA vaccines including mHSP70 gene did not improve the efficiency of SjC23 vaccines, which is not consistent with previously published studies [35]. It might be due to the small sample, or mHSP70 may play different role in different vaccines. The reason deserves further investigation.

In conclusion,vaccination using the DNA vaccine with the novel delivery vector-PAMAM-Lys provided 45%–50% worm reductions and 59%–62% liver egg reductions, which were dramatically higher than efficacy levels for naked DNA vaccines. As we know, in the field of vaccine research against schistosomiasis, the 50–60% improvement in protection in a mouse model as measured by reductions in adult worm and liver egg burden via a single antigen-based vaccine is very encouraging. Therefore, DNA vaccines with the novel delivery vector produced acceptable protective efficacy levels in a mouse model. Futhermore, in this study, we demonstrated that PAMAM-Lys dendrimers, as a novel vaccine delivery vector, may enhance the immunoreactivity of the DNA vaccine, and increase the protective effects of the SjC23 DNA vaccine against *S.japonicum* infection. Our findings suggest that this novel vaccine delivery vector may provide an efficient and safe delivery vector for *S.japonicum* vaccine research, and may also be used for other vaccines designs in the future.
